# Characterization of Direct Perturbations on Voltage-Gated Sodium Current by Esaxerenone, a Nonsteroidal Mineralocorticoid Receptor Blocker

**DOI:** 10.3390/biomedicines9050549

**Published:** 2021-05-13

**Authors:** Wei-Ting Chang, Sheng-Nan Wu

**Affiliations:** 1 Department of Biotechnology, Southern Taiwan University of Science and Technology, Tainan 71005, Taiwan; cmcvecho2@gmail.com; 2 Division of Cardiovascular Medicine, Chi-Mei Medical Center, Tainan 71004, Taiwan; 3 Institute of Clinical Medicine, College of Medicine, National Cheng Kung University, Tainan 70101, Taiwan; 4 Department of Physiology, National Cheng Kung University Medical College, Tainan 70101, Taiwan; 5 Institute of Basic Medical Sciences, National Cheng Kung University Medical College, Tainan 70101, Taiwan

**Keywords:** esaxerenone, voltage-gated Na^+^ current, persistent Na^+^ current, current kinetics, hysteresis, pituitary cell

## Abstract

Esaxerenone (ESAX; CS-3150, Minnebro^®^) is known to be a newly non-steroidal mineralocorticoid receptor (MR) antagonist. However, its modulatory actions on different types of ionic currents in electrically excitable cells remain largely unanswered. The present investigations were undertaken to explore the possible perturbations of ESAX on the transient, late and persistent components of voltage-gated Na^+^ current (*I*_Na_) identified from pituitary GH_3_ or MMQ cells. GH_3_-cell exposure to ESAX depressed the transient and late components of *I*_Na_ with varying potencies. The IC_50_ value of ESAX required for its differential reduction in peak or late *I*_Na_ in GH_3_ cells was estimated to be 13.2 or 3.2 μM, respectively. The steady-state activation curve of peak *I*_Na_ remained unchanged during exposure to ESAX; however, recovery of peak *I*_Na_ block was prolonged in the presence 3 μM ESAX. In continued presence of aldosterone (10 μM), further addition of 3 μM ESAX remained effective at inhibiting *I*_Na_. ESAX (3 μM) potently reversed Tef-induced augmentation of *I*_Na_. By using isosceles-triangular ramp pulse with varying durations, the amplitude of persistent *I*_Na_ measured at high or low threshold was enhanced by the presence of tefluthrin (Tef), in combination with the appearance of the figure-of-eight hysteretic loop; moreover, hysteretic strength of the current was attenuated by subsequent addition of ESAX. Likewise, in MMQ lactotrophs, the addition of ESAX also effectively decreased the peak amplitude of *I*_Na_ along with the increased current inactivation rate. Taken together, the present results provide a noticeable yet unidentified finding disclosing that, apart from its antagonistic effect on MR receptor, ESAX may directly and concertedly modify the amplitude, gating properties and hysteresis of *I*_Na_ in electrically excitable cells.

## 1. Introduction

Esaxerenone (ESAX; Minnebro^®^, CS-3150, XL-560, Oklahoma, Japan), known to be a newly oral, non-steroidal selective blocker on the activity of mineralocorticoid receptor (MR), has been growingly used for the management of varying pathologic disorders, such as primary aldosteronism, refractory hypertension, chronic kidney disease, diabetic nephropathy, and heart failure [1,2,3,4,5,6,7,8,9,10]. Alternatively, the activity of MR has been previously reported in pituitary cells including GH_3_ cells or in varying brain regions [11,12,13,14,15,16,17,18]. However, whether ESAX exercises any perturbations on electrical activities (e.g., transmembrane ionic currents) in pituitary cells is largely unknown.

There are nine isoforms (i.e., Na_V_1.1–1.9 [or SCN1A-SCN5A and SCN8A-SCN11A]) which are expressed in mammalian excitable tissues, including endocrine system [19,20]. Of notice, several inhibitors have been previously described to preferentially block the late component of voltage-gated Na^+^ current (*I_Na_*), such as ranolazine (Ran) and KMUP-1 [21,22], while some of Na_V_-channel activators (e.g., pyrethroids and telmisartan) have been reported in different types of excitable cells [23,24,25,26]. As an endocrine-disrupting agent, tefluthrin or cypermethrin is, respectively, type I or II pyrethroid known to activate *I*_Na_ [23,25,27]. The *I*_Na_ can be readily evoked in response to changes in the membrane potential sensed by the channel’s voltage-sensor domains, which are intimately coupled to its pore domain [28,29,30,31]. However, at present, whether and how ESAX is capable of interacting directly with Na_V_ channels to perturb the magnitude, gating properties, or hysteretic strength of *I*_Na_ in electrically excitable cells remains little thoroughly reported.

In view of the aforesaid considerations, we sought to explore whether there are any possible modifications of ESAX on the amplitude, kinetics, and hysteretic behavior of voltage-gated Na^+^ current (*I*_Na_) in electrically excitable cells (e.g., pituitary GH_3_ and MMQ cells). The present study, for the first time, demonstrated that, despite its effectiveness in antagonizing MR activity, the distinguishable inhibition by ESAX of peak and late *I*_Na_ may be caused by one of several ionic mechanisms underlying its marked perturbations on the functional activities of electrically excitable cells, assuming that similar observations exist in vivo.

## 2. Materials and Methods

### 2.1. Chemicals, Drugs, and Solutions Used in This Study

Esaxerenone (ESAX; Minnebro^®^ CS-3150, XL-550, 1-(2-hydroxyethyl)-4-methyl-N-(4-methylsulfonylphenyl)-5-[2-(trifluoromethyl)phenyl]pyrrole-3-carboxamide, C_22_H_21_F_3_N_2_O_4_S, https://pubchem.ncbi.nlm.nih.gov/compound/Esaxerenone, accessed in Oklahoma, Japan, on 26 February 2019) was acquired from MedChemExpress (Genechain, Kaohsiung, Taiwan), aldosterone (Aldo), dexamethasone (Dex), tefluthrin (Tef), tetraethylammonium chloride (TEA), and tetrodotoxin (TTX) were from Sigma-Aldrich (Merck Ltd., Taipei, Taiwan), while ranolazine (Ran) was from Tocris (Union Biomed, Taipei, Taiwan). Unless otherwise stated, culture media (e.g., F-12 medium), horse or fetal bovine serum, L-glutamine, and trypsin/EDTA were purchased from HyClone^TM^ (Thermo Fisher Scientific, Kaohsiung, Taiwan), while all other chemicals, such as aspartic acid, CsOH, CsCl, EGTA, and HEPES, were of laboratory grade and taken from standard sources.

The HEPES-buffered normal Tyrode’s solution used in this investigation had an ionic composition, which contained (in mM): NaCl 136.5, CaCl_2_ 1.8, KCl 5.4, MgCl_2_ 0.53, glucose 5.5, and HEPES 5.5, and the solution pH was titrated to 7.4 by adding NaOH. For measurements of *I*_Na_, we kept GH_3_ or MMQ cells bathed in Ca^2+^-free, Tyrode’s solution in order to preclude the contamination of Ca^2+^-activated K^+^ and voltage-gated Ca^2+^ currents. To record K^+^ currents, the solution used to fill up the recording electrode contained (in mM): K-aspartate 130, KCl 20, KH_2_PO_4_ 1, MgCl_2_ 1, Na_2_ATP 3, Na_2_GTP 0.1, EGTA 0.1, HEPES 5, and the solution was adjusted with KOH to pH 7.2. To record *I*_Na_ or *I*_Na(P)_, we substituted K^+^ ions in the internal solution for equimolar Cs^+^ ions and the pH value in the solution was then titrated to 7.2 by adding CsOH. All solutions used in this work were prepared using demineralized water from Mill-Q purification system (Merck Ltd., Taipei, Taiwan). On the day of experiments, we filtered the bathing or filling solution and culture medium by using an Acrodisc^®^ syringe filter with a 0.2-μm pore size (Bio-Check, New Taipei City, Taiwan).

### 2.2. Cell Preparations

The pituitary adenomatous cell line (GH_3_) was acquired from the Bioresource Collection and Research Center (BCRC-60015, http://catalog.bcrc.firdi.org.tw/Bcrc-Content?bid=60015, accessed on 4 April 2012, Hsinchu, Taiwan) [32], while the MMQ cell line (ATCC^®^ CRL-10609, https://www.atcc.org/products/all/CRL-10609.aspx, accessed on 18 April 2019), another established pituitary cell line originally derived from *Rattus norvegicus* pituitary prolactinoma, was from the American Type Cell Collection (Manassas, VA, USA) through Genechain (Kaohsiung, Taiwan). GH_3_ cells were maintained in Ham’s F-12 culture medium supplemented with 2.5% fetal bovine serum (*v/v*), 15% horse serum (*v/v*), and 2 mM L-glutamine [31], and MMQ cells were cultured in F12 medium supplemented with 10% fetal bovine serum (*v/v*) and 1.176 g/L NaHCO_3_ in a humidified incubator at 30 °C with 5% CO_2_ [33]. The cells were grown as a monolayer culture in a humidified environment of 5% CO_2_/95% air at 37 °C. The electrophysiological recordings were commonly made five or six days after the cells had been cultured to 60–80% confluence.

### 2.3. Electrophysiological Measurements

On the day of the experiments, we gingerly dispersed GH_3_ or MMQ cells with a 1% trypsin-EDTA solution, and a few drops of cell suspension was thereafter placed in a home-made chamber affixed on the stage of an inverted DM-II microscope (Leica; Major Instruments, Kaohsiung, Taiwan). Cells were kept immersed at room temperature (20–25 °C) in normal Tyrode’s solution, the composition of which was provided above, and they were allowed to settle on the chamber’s bottom. The patch electrodes were drawn from 1.8-mm OD round borosilicate glass tubing (Kimax-51 [#34500]; Kimble; Dogger, New Taipei City, Taiwan) using a two-stage pull on a vertical pipet puller (PP-83; Narishige; Major Instruments, Tainan, Taiwan). The recording electrodes used had a tip diameter of ~1 μm and a resistance of 3–5 MΩ. They were mounted in an air-tight holder, having a suction port on the side, and a chloride silver wire was used to make contact with the electrode solution. We recorded different types of ionic currents in the whole-cell mode of standard patch-clamp technique with dynamic adaptive suction (i.e., a decremental change in the suction pressure in response to progressive increase in the seal resistance) with the aid of either an Axoclamp-2B (Molecular Devices, Sunnyvale, CA, USA) or an RK-400 amplifier (Bio-Logic, Claix, France) [23,32]. This procedure was noticed to increase the success rate of forming gigaseals and reduced loss of the gigaseal in long-term recordings. For whole-cell current recordings, following seal formation (>1 GΩ) the membrane was ruptured by gentle suction. The liquid junction potentials were zeroed immediately before seal formation was made, and the whole-cell data were corrected.

### 2.4. Potential and Current Recordings

The signals were monitored and stored online at 10 kHz in an ASUS VivoBook Flip 14 laptop computer (TP412FA-0131A10210U; ASUS, Tainan, Taiwan) equipped with a Digidata 1440A interface (Molecular Devices). During the measurements with analog-to-digital and digital-to-analog conversion, the latter device was controlled using pCLAMP v10.7 software (Molecular Devices) run under Microsoft Windows 10 (Redmond, WA, USA). The laptop computer was put on the top of an adjustable Cookskin stand (Ningbo, Zheijiang, China) to allow efficient manipulation during the recordings. Through digital-to-analog conversion, the pCLAMP-created voltage-clamped protocols with varying rectangular or ramp waveforms were specifically designed and suited for determining the steady-state or instantaneous relationship pf current versus voltage (*I–V*) and voltage-dependent hysteresis of the current involved.

### 2.5. Data Analyses

The concentration-response data for inhibition of transient (peak) or late *I*_Na_ in pituitary GH_3_ cells were appropriately fitted to the Hill equation. That is,

(1) $$\%\;\mathrm{decrease}=\frac{{\left[ESAX\right]}^{n_{H}}\times E_{max}}{IC_{50}^{n_{H}}+{\left[ESAX\right]}^{n_{H}}}$$

In this equation, *[ESAX]* is the *ESAX* concentration applied, *n_H_* the Hill coefficient, *E_max_* the maximal inhibition of transient (peak) or late *I*_Na_ by *ESAX*, and *IC*_50_ the concentration required for a 50% inhibition of transient (peak) or late *I*_Na_.

The steady-state activation curve of peak *I*_Na_ with or without the *ESAX* addition observed in GH_3_ cells was established on the basis of a simple Boltzmann distribution (or the Fermi-Diract distribution) [31]:

(2) $$G_{Na}=\frac{G_{Na\left(max\right)}}{1+e{\frac{-\left(V-V_{\frac{1}{2}}\right)qF}{RT}}^{}}$$

where *G*_Na(max)_ is the maximal conductance of peak *I*_Na_ in the absence or presence of 3 μM *ESAX*; *V* the membrane potential in mV; *V*_1/2_ the half-point of the relationship; *q* the apparent gating charge; *F* Faraday’s constant; *R* the universal gas constant; *T* the absolute temperature, and *RT*/*F* = 25.4 mV.

### 2.6. Statistical Analyses

Curve-fitting (linear or non-linear) to experimental data sets was performed with the goodness of fit by using various maneuvers such as Microsoft Solver function embedded in Excel 2016 (Microsoft) and 64-bit OriginPro^®^2016 program (OriginLab; Scientific Formosa, Kaohsiung, Taiwan). The averaged results are presented as the mean ± standard error of the mean (SEM), with sample sizes (n) indicative of the number of GH_3_ or MMQ cells from which the experimental data were collected together. The Student’s *t*-test (paired or unpaired) and a one-way analysis of variance (ANOVA) followed by post-hoc Fisher’s least-significance difference method, were implemented for the evaluation of differences among means. Statistical significance was determined at a *p*-value of <0.05, unless otherwise indicated.

## 3. Results

### 3.1. Effect of Esaxerenone (ESAX) on Voltage-Gated Na^+^ Current (I_Na_) Measured from GH_3_ Cells

**Figure 1:**
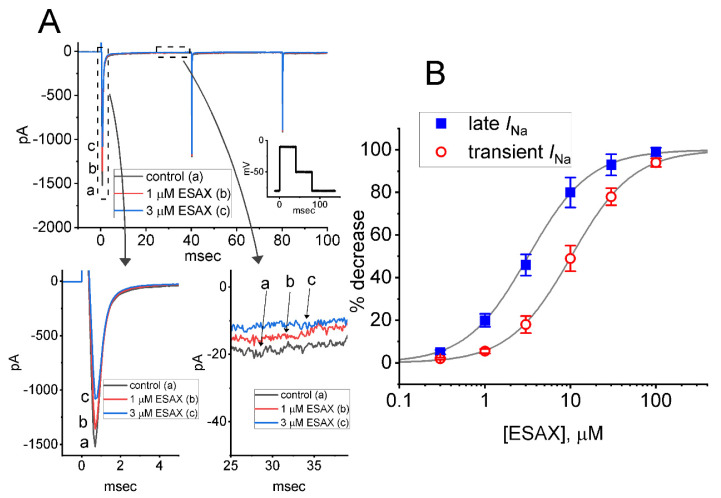
Effect of esaxerenone (ESAX) on voltage-gated Na^+^ current (*I*_Na_) measured from pituitary tumor (GH_3_) cells. This set of experiments was undertaken in cells bathed in Ca^2+^-free, Tyrode’s solution containing 10 mM TEA, and we filled up the electrode by using Cs^+^-containing solution. (**A**) Representative *I*_Na_ traces obtained in (a) the control situation (i.e., ESAX was not present) and during cell exposure to 1 μM ESAX (b) or 3 μM ESAX (c). The voltage pulse protocol applied is shown in Inset. The lower panels in (**A**) show the expanded records from each dashed box. (**B**) Concentration-dependent relationship of ESAX on transient (or peak) (○) and late (■) component of *I*_Na_ evoked by abrupt membrane depolarization (mean ± SEM; *n* = 8 for each point). Each smooth line indicates the goodness-of-fit to the Hill equation, as described in Materials and Methods.

In the first stage of the whole-cell recordings, we measured the effects of ESAX on the amplitude and gating kinetics of *I*_Na_ activated by rapid membrane depolarization. We kept cells immersed in Ca^2+^-free, Tyrode’s solution containing 10 mM tetraethylammonium chloride (TEA), and the recording electrode was filled up with a Cs^+^-containing solution. As demonstrated in Figure 1A, 1 min after GH_3_-cells were exposed to ESAX at a concentration of 1 or 3 μM, the amplitude in the peak (i.e., initial) or late (i.e., end-pulse) components of *I*_Na_ by abrupt depolarizing pulse from −80 to −10 mV was progressively depressed. For example, as the rectangular voltage step from −80 to −10 mV with a duration of 40 ms was delivered (indicated in the inset of Figure 1A) to activate *I*_Na_, the addition of 3 μM ESAX was noticed to result in an evident decrease in the peak or late amplitude of *I*_Na_ to 1104 ± 197 or 9 ± 1 pA (*n* = 8, *p* < 0.05) from control values of 1498 ± 241 or 19 ± 3 pA (*n* = 8), respectively. After removal of ESAX, the peak or late amplitude of the current returned to 1452 ± 228 or 18 ± 3 pA (*n* = 8, *p* < 0.05), respectively. Moreover, as cells were exposed to 1 μM tetrodotoxin (TTX) alone, peak amplitude of *I*_Na_ evoked by the same voltage protocol was almost abolished, as evidenced by a reduction of current amplitude to 54 ± 6 pA (*n* = 7, *p* < 0.01) from a control value of 1486 ± 235 pA (*n* = 7).

Figure 1B shows that the presence of ESAX to the bath can concentration-dependently decrease the amplitude of peak or late *I*_Na_ evoked by rapid depolarizing pulse. According the Hill equation detailed under Materials and Methods, the IC_50_ value needed for ESAX-mediated block of peak or late *I*_Na_ detected in GH_3_ cells was yielded to be 13.2 or 3.2 μM, respectively, the value of which was apparently distinguishable between its suppressive effects on these two components of the current. Our initial experiments; therefore, enable us to reflect that ESAX exercises a depressant action on the peak and late *I*_Na_ natively expressed in GH_3_ cells, and that this drug tends to be selective for later over peak *I*_Na_ in response to rectangular depolarizing step.

### 3.2. Effect of ESAX on the Steady-State Current-Voltage (I–V) or Conductance-Voltage Relationship of Peak I_Na_

**Figure 2:**
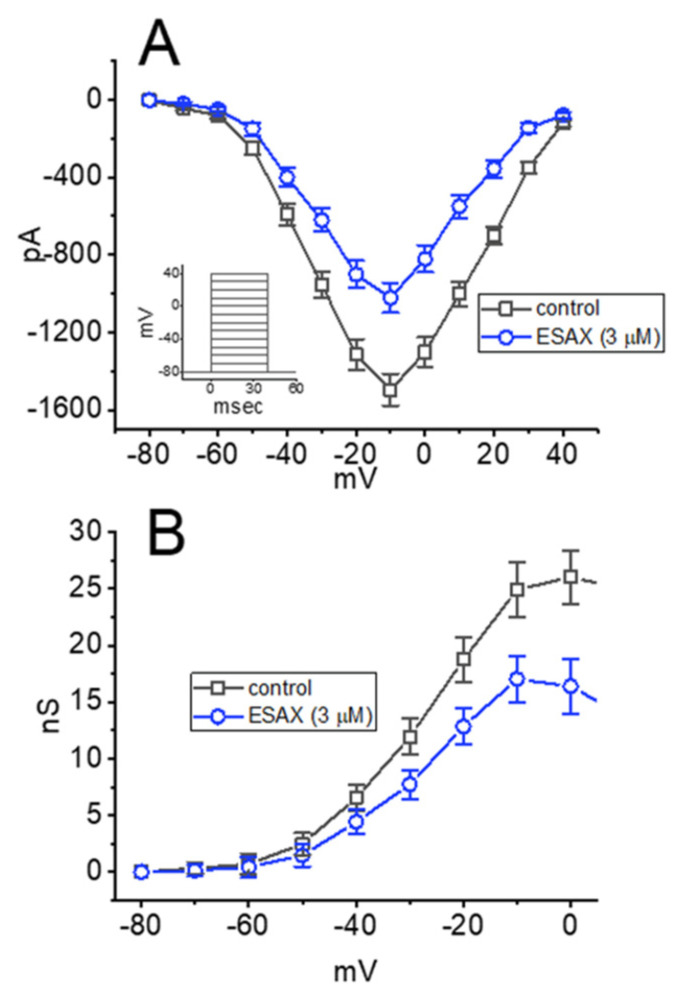
Effect of ESAX on current versus voltage (*I–V*) and conductance versus voltage relationships of *I*_Na_ identified in GH_3_ cells. (**A**) Mean *I–V* relationship of peak *I*_Na_ taken without (□) or with (○) the addition of 3 μM ESAX (mean ± SEM; *n* = 7 for each point). Inset shows the voltage-clamp protocol delivered. (**B**) Mean conductance versus voltage relationship of peak *I*_Na_ taken in the control period (□) and during cell exposure (○) to 3 μM ESAX (mean ± SEM; *n* = 7 for each point). Of notice, the conductance versus voltage relationship of the current between the absence and presence of ESAX did not differ, despite the decrease in maximal conductance of peak *I*_Na_.

To characterize the inhibitory effect of ESAX on *I*_Na_, we next examined whether this drug might exercise any perturbations on the steady-state *I–V* relationship of *I*_Na_ in GH_3_ cells. Figure 2A illustrates the mean *I–V* relationship of peak *I*_Na_ with or without the addition of 3 μM ESAX. It was noticed that the value of threshold or maximal voltage for the elicitation of *I*_Na_ did not differ between the absence and presence of 3 μM ESAX. The relationship of *I*_Na_-conductance versus membrane potential obtained with or without the application of 3 μM ESAX was also established and depicted in Figure 2B. Similar experimental results were obtained in seven different cells examined. The data; thus, led us to reflect that no obvious change in the steady-state activation curve of peak *I*_Na_ in GH_3_ cells was detected during exposure to ESAX.

### 3.3. Effect of ESAX on the Recovery of I_(Na)_ Block Recorded from GH_3_ Cells

**Figure 3:**
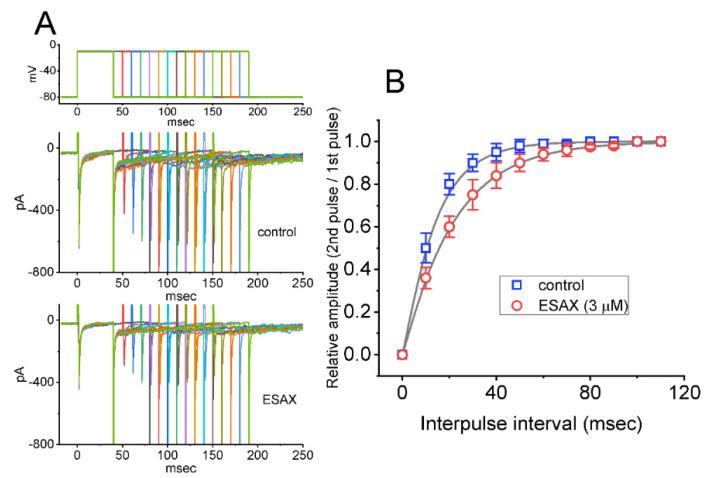
ESAX-induced prolongation in the recovery of *I*_Na_ block in GH_3_ cells. Cells were bathed in Ca^2+^-free, Tyrode’s solution, the electrode was filled with Cs^+^-containing solution, and a set of two-pulse voltage protocol was applied to the examined cells. (**A**) Representative current traces showing current recovery from block by use of two-step protocol with varying durations (as indicated in the uppermost part). Current traces in the upper part were obtained in the control period, while those in the lower part were taken during cell exposure to 3 μM ESAX. (**B**) Relationship of relative amplitude versus interpulse interval in the absence (□) and presence (○) of 3 μM ESAX. The relative amplitude of peak *I*_Na_ was taken by the ratio of current amplitudes elicited by the second depolarizing voltage step and those by the first pulse. Each point represents the mean ± SEM (*n* = 8). The smooth line indicates a best fit to single exponential.

In an effort to evaluate ESAX-induced inhibition of peak *I*_Na_ in these cells, we continued to investigate the recovery from current inactivation in the absence and presence of this drug. In this set of experiments, a stimulation protocol consisting of a first (conditioning) depolarizing voltage command which is sufficiently long to reach a steady-state was employed, and a second depolarizing (test pulse) was applied at the same potential as the conditioning pulse (Figure 3). Then the ratios of the peak I_Na_ in response to the second and first pulses were taken as a measure of recovery from I_(Na)_ block, and they were thereafter established and plotted versus interpulse interval. The *I*_Na_ recovery from block with or without the application of 3 μM ESAX is illustrated in Figure 3. In the absence or presence of 3 μM ESAX, the time course could be described by single exponential with the time constants of 13.2 ± 2.1 ms (*n* = 8) or 21.5 ± 2.7 ms (*n* = 8), respectively. It is; thus, plausible to anticipate, from the present results, that the recovery of *I*_Na_ block was prolonged by the presence of ESAX, and that the delayed recovery caused by this agent may ascribe largely from the open channel block.

### 3.4. Comparisons among Effect of ESAX, ESAX plus Ranolazine (Ran), ESAX plus Dexamethasone (Dex), Aldosterone (Aldo), and Aldosterone plus ESAX on Peak I_Na_ Recorded from GH_3_ Cells

**Figure 4:**
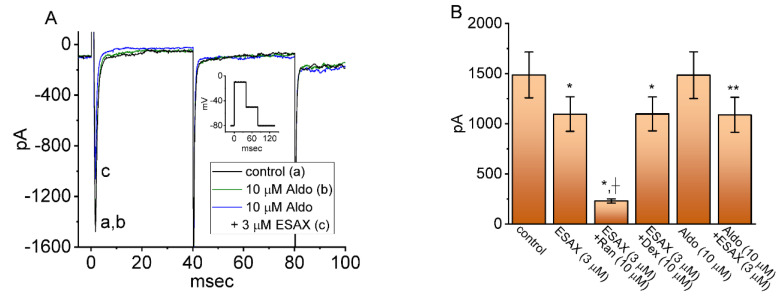
Comparison among effects of ESAX, ESAX plus ranolazine (Ran), aldosterone (Aldo), and aldosterone plus ESAX on the amplitude of peak *I*_Na_ detected in GH_3_ cells. Cells were kept bathed in Ca^2+^-free, Tyrode’s solution, and the recording electrode was filled up with Cs^+^-containing solution. The *I*_Na_ was elicited by 40 ms depolarizing voltage command from −80 to −10 mV, and current amplitude at the start of the voltage pulse was measured. (**A**) Representative current traces obtained in the control (a) and during cell exposure to 10 μM aldosterone (Aldo) (b) or 10 μM Aldo plus 3 μM ESAX (c). Inset shows the voltage pulse protocol. (**B**) Summary bar graph showing effects of ESAX, ESAX plus Ran, ESAX plus Dex, Aldo, and Aldo plus ESAX. Each bar represents the mean ± SEM (*n* = 7–8). * Significantly different from control (i.e., none of the agents were present) (*p* < 0.05), + significantly different from ESAX (3 μM) alone group (*p* < 0.05), and ** significantly different from aldosterone (10 μM) alone group (*p* < 0.05).

In another series of whole-cell experiments, we tested the possible effects of ESAX, ESAX plus Ran, ESAX plus Dex, Aldo, and Aldo plus ESAX on the peak amplitude of *I*_Na_ activated in response to abrupt membrane depolarization from −80 to −10 mV. Ranolazine has been demonstrated to inhibit *I_Na_* potently [21,32], while Dex could perturb membrane ion currents in pituitary cells or bronchial smooth myocytes [34,35]. The expression of mineralocorticoid receptor (MR) has been shown previously to be expressed in GH_3_ cells [11,12,18,36,37]. As shown in Figure 4A, Aldo (10 μM) alone did not modify peak *I*_Na_; however, further addition of ESAX (3 μM), but still in the presence of Aldo, decreased current amplitude. As summarized in Figure 4B, ESAX at a concentration of 3 μM produced an inhibitory effect on peak *I*_Na_ in GH_3_ cells; moreover, the subsequent addition of 10 μM ranolazine, still in the presence of 3 μM ESAX, was effective at decreasing *I*_Na_ amplitude further. However, as cells were continually exposed to 3 μM ESAX, further application of 10 μM Dex failed to modify its decrease in the peak *I*_Na_. On the other hand, the presence of 10 μM Aldo alone did not produce any appreciable changes in the amplitude of peak *I*_Na_; however, subsequent addition of 3 μM ESAX could reduce current amplitude further. Hence, its seems that Dex or Aldo did not exert any effect on ESAX-mediated decrease of peak *I*_Na_.

### 3.5. Enhanced Amplitude by Tef of I_Na_ Attenuated by ESAX

**Figure 5:**
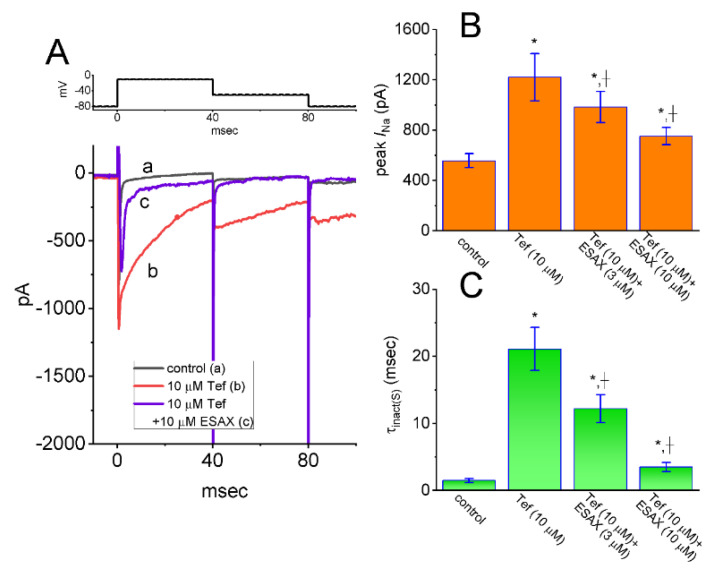
Effect of ESAX on tefluthrin (Tef)-mediated augmentation of *I*_Na_ recorded from GH_3_ cells. (**A**) Representative current traces activated by rapid membrane depolarization (indicated in the upper part). Current trace labeled a is control, that labeled b was taken during exposure to 10 μM Tef, and that labeled **c** was obtained in the presence of 10 μM Tef plus 10 μM ESAX. The upper part in (**A**) shows the voltage-clamp protocol applied. Summary bar graphs shown in (**B**,**C**) respectively demonstrate effects of Tef or Tef plus ESAX on peak *I*_Na_ and the time constant (τ_inact(S)_) in the slow component of *I*_Na_ inactivation (mean ± SEM; *n* = 7 for each bar). * Significantly different from controls (*p* < 0.05), and + significantly different from Tef (10 μM) alone group (*p* < 0.05).

As an endocrine disrupting action, pyrethroids such as cypermethrin have been reported to increase the plasma level of hormones including Aldo [27]. We, hence, next investigated whether the presence of ESAX could modify Tef-enhanced *I*_Na_ evoked in response to rapid depolarizing pulse in GH_3_ cells. In these experiments, when whole-cell current recordings were established, we maintained the examined cell in voltage clamp at −80 mV and the 40 ms depolarizing pulse to −10 mV was applied to it. Of notice, in keeping with previous observations [23,25,32], 1 min of exposing cells to 10 μM Tef alone, the peak amplitude of *I*_Na_ measured at the start of depolarizing pulse was enhanced, in combination with a measurable slowing in the inactivation or deactivation rate of the current. Moreover, subsequent addition of EASX at a concentration of 3 or 10 μM could attenuate Tef-mediated augmentation on depolarization-activated *I*_Na_ (Figure 5). For example, as the cells were depolarized from −80 to −10 mV to evoke *I*_Na_, the exposure to 10 μM Tef resulted in a significant raise in peak *I*_Na_ amplitude from 557 ± 56 to 1221 ± 187 pA (*n* = 7, *p* < 0.05). The further application of 3 μM ESAX, still in the continued presence of Tef, greatly reduced to 984 ± 123 pA (*n* = 7, *p* < 0.05). Again, the presence of 10 μM Tef evidently increased the time constant (τ_inact(S)_) in the slow component of *I*_Na_ inactivation from 1.5 ± 0.3 to 21.1 ± 3.2 ms (*n* = 7, *p* < 0.01), while subsequent addition of 3 μM ESAX resulted in a significant decline in the τ_inact(S)_ value to 12.2 ± 2.1 ms (*n* = 7, *p* < 0.05). In this regard, the data reflected that the presence of ESAX was capable of reversing Tef-mediated enhancement in both amplitude and τ_inact(S)_ of peak *I*_Na_ observed in GH_3_ cells.

### 3.6. Augmented Amplitude and Hysteresis by Tef of Persistent Na^+^ Current (I_Na(P)_) Attenuated by ESAX

**Figure 6:**
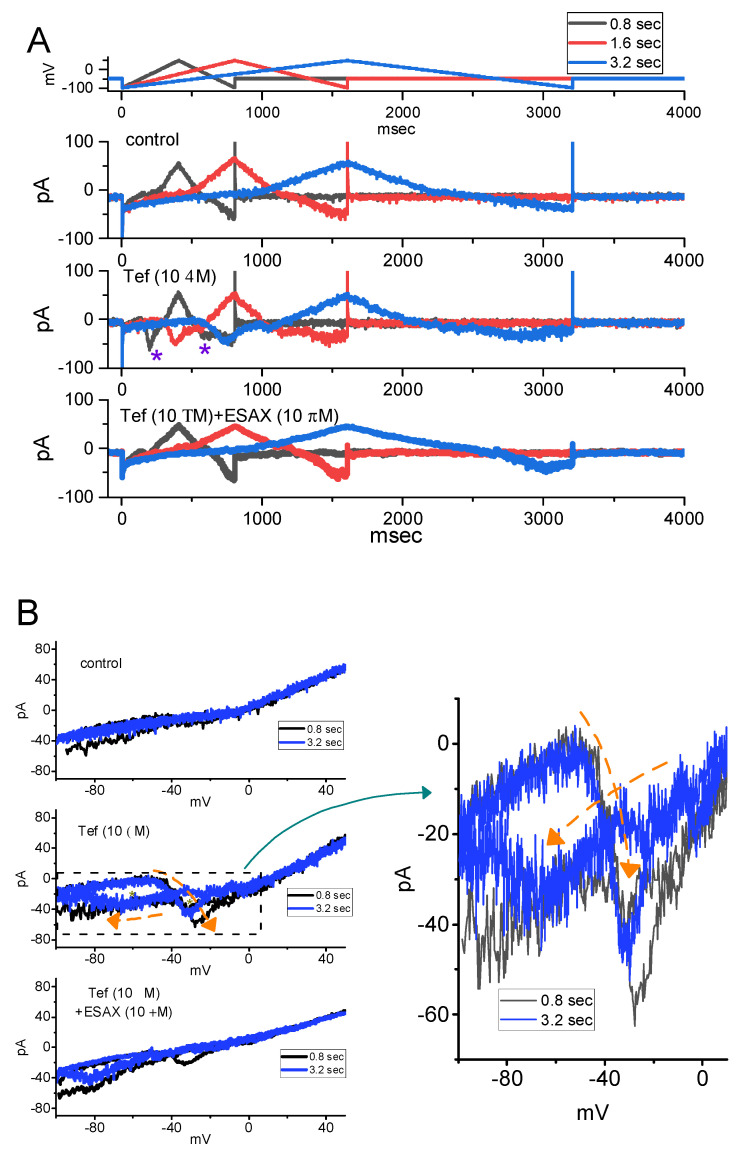

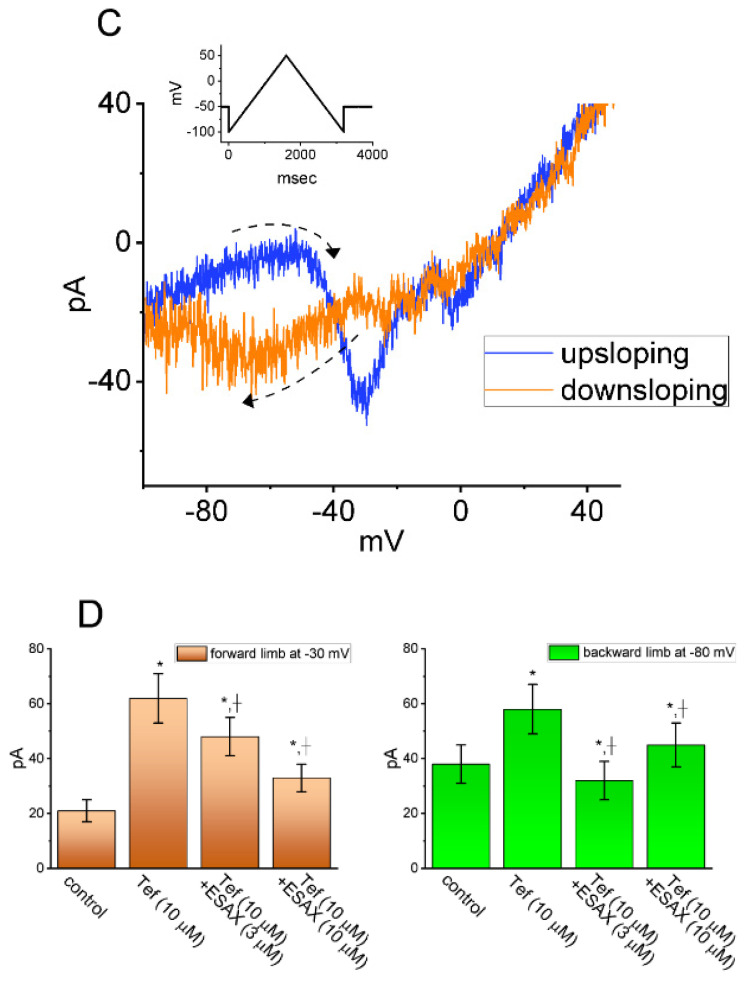
Inhibitory effect of ESAX on Tef-mediated increase in persistent *I*_Na_ (*I*_Na(P)_) activated by isosceles-triangular ramp pulse in GH_3_ cells. In these whole-cell current recordings, the potential applied to the examined cell was held at −50 mV and the isosceles-triangular ramp voltage with varying durations of 0.4 to 3.2 s (i.e., ramp speed of ± 0.094 to 0.75 to mV/ms) to activate *I*_Na(P)_ in response to the forward (from −100 to +50 mV) and backward (from +50 to −100 mV) ramp voltage-clamp command was delivered to it. (**A**) Representative current traces obtained in the control period (upper), and during cell exposure to Tef (10 μM) (middle), or to Tef (10 μM) plus ESAX (10 μM) (lower). The black, red, or blue color indicated in the right upper side respectively denote the duration of isosceles-triangular ramp pulse applied (i.e., 0.8, 1.6 or 3.2 s, (i.e., the ramp speed of ±0.38, ±0.19 or ±0.094 mV/ms)). Purple asterisks in the middle part of (**A**) shows Tef-induced augmentation in the amplitude of *I*_Na(P)_ elicited by the upsloping and downsloping ends of the triangular ramp. (**B**) Representative instantaneous *I–V* relation of *I*_Na(P)_ in response to isosceles-triangular ramp pulse with a duration of 0.8 s (black color) or 3.2 s (blue color). The current traces in the upper part are controls, while those in the middle or lower part were respectively acquired from the presence of Tef (10 μM) alone or Tef (10 μM) plus ESAX (10 μM). The dashed orange arrows in the middle part and the right side of (**B**) show the direction of *I*_Na(P)_ trajectories in which time passes during the elicitation by the upright isosceles-triangular ramp pulse with a duration of 0.8 s (black color) or 3.2 s (blue color). The graph in the right side shows an expanded record of the dashed box in the left middle part. The asterisks in the middle part of (**B**) indicate the figure-of-eight (or infinity-shaped: ∞) loop (as demonstrated in the right side) of voltage-dependent hysteresis responding to the triangular ramp. (**C**) Figure-of-eight pattern in voltage-dependent hysteresis of *I*_Na(P)_ activated by isosceles-triangular ramp voltage with a ramp duration of 3.2 s (or a ramp speed of ±0.094 mV/ms) in the presence of 10 μM Tef. The ascending limb is indicated in blue color, while the descending one is in the orange color. The dashed arrow indicates the direction of current trajectory by which time goes. (**D**) Summary bar graph showing the effects of Tef (10 μM) and Tef (10 μM) plus ESAX (10 μM) on *I*_Na(P)_ amplitude activated by the upsloping and downsloping limb of 0.8-s triangular ramp pulse (mean ± SEM; *n* = 7 for each bar). Current amplitude in the left side was taken at the level of −30 mV in situations where the forward (upsloping) limb of triangular pulse was applied to evoke *I*_Na(P)_ (i.e., high-threshold *I*_Na(P)_), while that in the right side (i.e., low-threshold *I*_Na(P)_) was at −80 mV during the backward (downsloping) end of the pulse. * Significantly different from control (*p* < 0.05) and + significantly different from Tef (10 μM) alone group (*p* < 0.05).

We next continued to investigate whether cell exposure to ESAX could modify the Tef-mediated enhancement of *I*_Na(P)_ activated in response to the isosceles-triangular ramp pulse in GH_3_ cells. In these whole-cell current recordings, the examined cell was voltage-clamped at −50 mV and a set of upright isosceles-triangular ramp pulses ranging between −100 and +50 mV with varying durations at a rate of 0.05 Hz was gingerly applied to it through digital-to-analog conversion (Figure 6A). In accordance with previous observations [25,38], when GH_3_ cells were exposed to 10 μM Tef alone, the amplitude of *I*_Na(P)_ at high or low threshold respectively activated by the upsloping (forward, ascending) or downsloping (backward, descending) end of upright triangular ramp voltage was raised and a striking figure-of-eight hysteresis (i.e., ∞) in the instantaneous *I–V* relationship of *I*_Na(P)_ was concurrently revealed (Figure 6B,C). For example, as the isosceles-triangular ramp pulse with a duration of 0.8 s (or ramp speed of ±0.38 mV/ms) was applied, in the presence of 10 μM Tef, the peak *I*_Na(P)_ amplitude measured at the level of −30 mV (i.e., high-threshold *I*_Na(P)_) during the ascending phase of triangular ramp pulse was conceivably raised to 62 ± 9 pA (*n* = 7, *p* < 0.05) from a control value (measured at the same level of membrane potential) of 21 ± 4 pA (*n* = 7). Meanwhile, during exposure to 10 μM Tef, the peak *I*_Na(P)_ amplitude measured at −80 mV during the descending phase of such pule was concurrently raised from 38 ± 7 to 58 ± 9 pA (*n* = 7, *p* < 0.05). In particular, as depicted in Figure 6B, the subsequent addition of 3 μM ESAX, but still in the presence of 10 μM Tef, was able to produce a progressive reduction in the amplitude of high- or low-threshold peak *I*_Na(P)_ measured at −30 or −80 mV to 48 ± 7 or 58 ± 9 pA (*n* = 7, *p* < 0.05), respectively. These observations; therefore, led us to indicate that the voltage dependence of *I*_Na(P)_ resulting in hysteretic changes observed in GH_3_ cells markedly emerged in the presence of Tef (Figure 6B,C). Further application of ESAX (3 or 10 μM) was noticed to attenuate Tef-induced increase in the respective high- and low-threshold amplitude of *I*_Na(P)_ activated by the ascending (rising) and descending (declining) phases of the isosceles-triangular ramp pulse detected in these cells (Figure 6D). It is conceivable; therefore, that the addition of ESAX, but still in the presence of Tef, resulted in a marked reduction in voltage-dependent hysteretic strength of the instantaneous *I–V* relationship of *I*_Na(P)_ in response to isosceles-triangular ramp voltage in GH_3_ cells.

### 3.7. Effect of ESAX on I_Na_ Identified in Pituitary MMQ Cells

**Figure 7:**
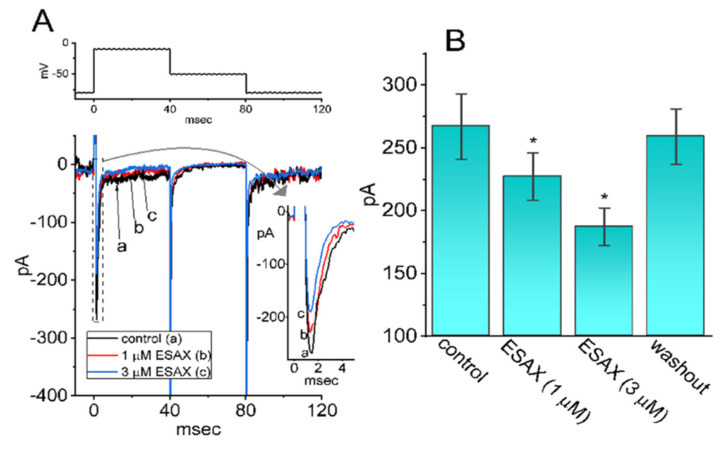
Effect if ESAX on *I*_Na_ is present in MMQ pituitary lactotrophs. In this series of experiments, we kept cells bathed in Ca^2+^-free Tyrode’s solution and the electrodes were backfilled with Cs^+^-containing solution. As whole-cell configuration was firmly established, we voltage-clamped the cell at −80 mV and the depolarizing voltage step to −10 mV followed by return to −50 mV was delivered to it. (**A**) Representative *I*_Na_ traces in response to depolarizing command pulses (indicated in the upper part). (a): control; (b): 1 μM ESAX; (c): 3 μM ESAX. Inset in the right side indicates an expanded record from dashed box. (**B**) Summary of the data showing effect of ESAX (1 or 3 μM) on peak amplitude of *I*_Na_ in MMQ cells (mean ± SEM; *n* = 8 for each bar). Current amplitude was measured at the start of 40 ms depolarizing pulse from −80 to −10 mV. * Significantly different from control (*p* < 0.05).

In another set of experiments, we tested whether *I*_Na_ present in another type of pituitary lactotrophs (i.e., MMQ cells) could still be modified by the presence of ESAX. The preparation on these cells was detailed under Materials and Methods. Cells were bathed in Ca^2+^-free, Tyrode’s solution containing 10 mM TEA and the pipet was filled up with Cs^+^-containing solution. Of notice, as MMQ cells were continually exposed to ESAX at a concentration of 1 or 3 μM, the amplitude of peak *I*_Na_ activated by abrupt 40 ms depolarization from −80 to −10 mV was appreciably diminished, in combination with the raised inactivation rate of the current (Figure 7). For example, cell exposure to 3 μM ESAX gradually decreased peak *I*_Na_ from 267 ± 26 to 187 ± 15 pA (*n* = 8, *p* < 0.05). After washout of the drug (i.e., ESAX was removed, but cells were still exposed to Ca^2+^-free, Tyrode’s solution containing 10 mM TEA), current amplitude returned to 259 ± 22 pA (*n* = 8, *p* < 0.05). Meanwhile, the τ_inact(S)_ value of the current was significantly shortened from 2.7 ± 0.8 to 1.7 ± 0.7 ms (*n* = 8, *p* < 0.05). Therefore, indistinguishable from the observations made in GH_3_ cells, the results that we have obtained showed the ability of ESAX to inhibit *I*_Na_ in response to the rapid depolarizing step in these cells.

## 4. Discussion

The principal findings in this study are that (a) the presence of ESAX could depress *I*_Na_ in a concentration, time-, state- and hysteresis-dependent manner identified in GH_3_ cells; (b) this drug resulted in the differential inhibition of peak or late amplitudes of *I*_Na_ activated by abrupt membrane depolarization with effective IC_50_ value of 13.2 or 3.2 μM, respectively; (c) ESAX did not modify the steady-state activation curve of peak *I*_Na_; however, the recovery of *I*_Na_ block was robustly prolonged in its presence; (d) the presence of Tef enhanced both the amplitude and τ_inact(S)_ of peak *I*_Na_, while, further addition of ESAX could effectively counteract Tef-induced modification of *I*_Na_; (e) subsequent application of ESAX was capable of depressing Tef-induced increase in the high- or low-threshold amplitude of *I*_Na(P)_ elicited by the isosceles-triangular ramp at either upsloping or downsloping limb, respectively; and (f) the presence of ESAX was effective at decreasing the amplitude and gating of *I*_Na_ in pituitary MMQ cells. Taken together with the present observations, the results allow us to reflect that ESAX-perturbed change in the amplitude, gating properties, and hysteretic behavior of *I*_Na_ tends to be independent and upstream of its action on the MR activity, and that it would hence conceivably participate in the adjustments on varying functional activities in electrically excitable cells (e.g., GH_3_ or MMQ cells) occurring in vivo.

In this study, the presence of neither Dex nor Aldo had minimal change on *I*_Na_. The addition of ESAX, but still in the continued presence of Aldo, was able to decrease the amplitude of peak *I*_Na_ in GH_3_ cells. Therefore, it seems unlikely that ESAX-mediated inhibition of the amplitude and gating kinetics of *I*_Na_ was largely associated with its blockade of MR. It is also important to be emphasized that the maximal plasma concentration of ESAX was reported previously to reach around 1416 ng/mL (or 3 μM) after 10-day administration with the dose of 100 mg/day [39]. As such, the inhibitory effects of ESAX on peak and late *I*_Na_ that we have obtained here could noticeably be of clinical or therapeutic relevance.

In the present investigations, the non-linear voltage-dependent hysteresis of *I*_Na(P)_ in the control period and during cell exposure to Tef or Tef plus ESAX was observed by the upright isosceles-triangular ramp voltage command with varying durations. In particular, as cells were exposed to 10 μM Tef, the peak *I*_Na(P)_ activated at the forward (upsloping) limb of the triangular ramp pulse with varying durations was noticed to be enhanced, particularly at the level of −30 mV, whereas the *I*_Na(P)_ amplitude at the backward (downsloping) end was increased at the level of −80 mV. Consequently, in the presence of 10 μM Tef, the figure-of-eight configuration in the hysteretic loop elicited by the triangular ramp pulse was evidently demonstrated (Figure 6). That is, there appeared to be the two types of *I*_Na(P)_, that is, low-threshold (i.e., activating at a voltage range near the resting potential) and high-threshold (i.e., activating at a voltage range near the maximal *I*_Na_ achieved) *I*_Na(P)_, observed during cell exposure to 10 μM Tef. The low-threshold *I*_Na(P)_ was noticeably activated (at the voltage range near the resting potential) upon the downsloping end of the triangular ramp pulse; however, the high-threshold (at the voltage range where peak *I*_Na_ was maximally activated) was by the upsloping end of such pulse. As the ramp speed decreased, the area of such hysteretic loop progressively reduced. Therefore, findings from the present observations revealed that the triangular pulse-induced *I*_Na(P)_ was observed to undergo hysteretic change in the voltage dependence found in GH_3_ cells [29,30,38].

In the present study, as GH_3_ cells were exposed to Tef, the voltage-dependent movement of S4 segment residing in Na_V_ channels could be perturbed; consequently, the coupling of the pore domain to the voltage-sensor domain was enhanced [25,27,29]. The voltage sensor energetically coupled to channel activation is supposed to be a conformationally flexible region of the protein. It is; therefore, tempting to speculate that such *I*_Na(P)_, particularly during exposure to Tef, could intrinsically and dynamically possess “memory” of previous or past events, or that mode shift occurs with respect to the voltage sensitivity of gating charge movement, which depends on the previous state (or conformation) of the Na_V_ channel [27,29,38]. Such unique type of hysteretic behavior inherently in Na_V_ channels would potentially play substantial role in influencing electrical behavior, Na^+^ overload due to an excessive Na^+^ influx, or hormonal secretion in different types of excitable cells during exposure to pyrethroid insecticides (e.g., tefluthrin or cypermethrin) [23,25,37,40]. Moreover, it needs to be noticed that the subsequent addition of 3 μM ESAX, still in the continued presence of 10 μM Tef, resulted in a considerable reduction of hysteretic strength responding to triangular ramp voltage.

In concert with its antagonistic action on MR, ESAX might exert an additional ameliorating action on salt-induced elevation of blood pressure or on kidney injuries [1,6,8,10,41,42]. ESAX has indeed reported the ability of alleviating effects in hypertensive patients with normal kidney function and in diabetic patients with albuminuria [5]. The activity of Na_V_ channels has been noticeably disclosed to be functionally expressed in varying types of vascular smooth muscle cells [19,32,43,44,45]. It was also shown that Ran, an inhibitor of late *I*_Na_ [21], could exercise certain beneficial action in the management of pulmonary hypertension [46]. The mRNA transcripts for the α-subunit of Na_V_1.1, Na_V_1.2, Na_V_1.3, and Na_V_1.6, as well as β1- and β3-subunits of Na_V_ channels, have been previously demonstrated in GH_3_ cell line [47]. Therefore, it is worth pursuing to a further extent as to which, despite its blockade on MR, ESAX-induced antihypertensive action is linked to its additional inhibitory action on *I*_Na_ (i.e., Na_V_1.7-encoded current) inherently in vascular smooth myocytes.

Finerenone, another structurally similar agent of ESAX, has been reported previously to perturb Aldo-dependent nuclear import of the MR [48]. The Aldo binding site or MR expression was demonstrated to be present in pituitary cells including GH_3_ cells [11,12,16,18,36]. However, the effects of ESAX on the amplitude, kinetics, and hysteretic strength of *I*_Na_ demonstrated in this study were found to be rapid and robust in onset; hence, such hastened action is expected to be independent of its action on the cytosolic activity of MR and it tends to occur in a non-genomic mechanism.

On the basis of the present observations, despite the antagonistic effect on MR [2,10], our results strongly imply that the inhibitory action of ESAX on transmembrane ionic channels, particularly on Na_V_ channels, tends to be direct obligate mechanisms. Through ionic mechanisms demonstrated herein, it or other structurally similar non-steroidal compounds (e.g., apararenone and finerenone), which can be preferentially used for oral intake, is able to adjust the functional activities of different types of endocrine or neuroendocrine cells, if similar in vivo findings exist. To this end, even during its perturbing action on the renin-angiotensin-Aldo system [5,6,42,49], findings from this study also highlight an important alternative aspect that needs to be taken into consideration, inasmuch as the beneficial effects of ESAX in different pathologic disorders, such as primary hyperaldosteronism, chronic kidney disease, refractory hypertension, and heart failure, have been currently evaluated [1,3,4,5,6,9,39,49,50,51].

## Data Availability

The original data is available upon reasonable request to the corresponding author.

## Abbreviations

Aldo, aldosterone; Dex, dexamethasone; ESAX, esaxerenone (1-(2-hydroxyethyl)-4-methyl-N-(4-methylsulfonylphenyl)-5-[2-(trifluoromethyl)phenyl]pyrrole-3-carboxamide); *I–V*, current versus voltage; IC_50_, concentration required for 50% inhibition; *I*_Na_, voltage-gated Na^+^ current; *I*_Na(P)_, persistent Na^+^ current; MR, mineralocorticoid receptor; Na_V_ channel, voltage-gated Na^+^ channel; Ran, ranolazine; SEM, standard error of mean; TEA, tetraethylammonium chloride; Tef, tefluthrin; TTX, tetrodotoxin; τ_inact(S)_, time constant in the slow component of current inactivation.
